# A Network Analysis of Food Intake and Cognitive Function in Older Adults with Multimorbidity: A National Cross-Sectional Study

**DOI:** 10.3390/nu17172767

**Published:** 2025-08-27

**Authors:** Xiyan Li, Chengyu Chen, Xinru Li, Xinyi Xu, Ting Zheng, Yuyang Li, Qinglei Cai, Huang Lin, Chichen Zhang

**Affiliations:** 1 School of Nursing, Southern Medical University, Guangzhou 510515, China; 18894136919@163.com (X.L.); chenchengyyu@163.com (C.C.); 2 School of Health Management, Southern Medical University, Guangzhou 510515, China; 17861521071@163.com (X.L.); xinyi_xu357@163.com (X.X.); zhti2002@163.com (T.Z.); liyuyang118@163.com (Y.L.); nc146@smu.edu.cn (Q.C.); 3 Key Laboratory of Philosophy and Social Sciences of Colleges and Universities in Guangdong Province for Collaborative Innovation of Health Management Policy and Precision Health Service, Guangzhou 510515, China; 4 School of Public Health, Southern Medical University, Guangzhou 510515, China; 5 Southern Medical University Center for Health Policy and Governance (Guangdong Provincial Social Science Research Base), Guangzhou 510515, China

**Keywords:** multimorbidity, cognitive function, food consumption, network analysis, health management

## Abstract

**Background:** Implementing effective interventions for specific cognitive symptoms is critical to reducing the disease burden of dementia. Previous studies have identified associations between overall cognitive function and dietary patterns in older adults with multimorbidity. However, the relationship between specific cognitive symptoms and different foods remains largely unknown. **Methods:** We included 3443 older adults with multimorbidity, aged 65 years or older, from the Chinese Longitudinal Health Longevity Survey (CLHLS, 2017–2018). We used the Chinese version of the Mini-Mental State Examination (MMSE) to assess cognitive function and selected 13 common foods to evaluate food consumption. Network analysis was used to identify central symptoms and bridge symptoms between the food consumption and cognitive symptom networks. Finally, the stability of the networks was examined using the case-dropping bootstrap procedure. **Results:** Network analysis revealed that B6 (mushrooms or algae), B4 (dairy products), and B5 (nut products) were the most influential in the food–cognition network model, and A5 (language ability), A1 (orientation ability), and B5 (nut products) were considered bridging symptoms in the food–cognition network. Bootstrap analysis showed that the 95% confidence interval of the edge weights in the network is narrow, indicating that this study accurately assesses the edge weights. The correlation stability coefficient of the centrality of the expected influence and bridge strength is 0.75, indicating that the network has good stability. **Conclusions:** Central symptoms as well as bridge symptoms play a key role in food and cognitive networks. Timely systematic and multilevel interventions targeting central symptoms and bridge symptoms may help to delay the risk of dementia in older adults with multimorbidity.

## 1. Introduction

As the population ages, the risk of cognitive impairment increases rapidly, and it has become a significant public health issue. Studies have indicated that over 360,000 individuals in China are diagnosed with cognitive impairment annually, and projections estimate that the total will reach 48.68 million by 2060 [1]. Cognitive impairment is characterized by an objective decline in at least one cognitive domain, including executive function, memory, attention, language, abstract reasoning, or visuospatial skills. Importantly, this condition serves as a critical precursor for dementia, substantially increasing the risk of its development [2]. Dementia is the most severe cognitive disorder, characterized by cognitive deficits that impair daily functioning and result in loss of independence. As a leading cause of disability and mortality among older adults, dementia places considerable personal, societal, and economic burdens [3]. Moreover, current therapeutic options are limited to symptomatic management, with no available disease-modifying agents to cure or slow the disease progression [4]. Consequently, identifying modifiable risk factors during the early stages of cognitive impairment, establishing targets for preventive interventions, and implementing precision strategies to delay or prevent dementia have become urgent public health priorities. These measures are critical for promoting successful aging and alleviating healthcare system burdens.

Multimorbidity, defined as the coexistence of two or more chronic conditions, is prevalent among the elderly [5]. Multimorbidity is associated with reduced physical function, reduced quality of life, and increased disability and mortality rates [6]. Research suggests that multimorbidity increases the risk for developing mild cognitive impairment (MCI) or dementia [7]. The progression of physical illnesses accelerates cognitive decline, particularly in the situational and working memory domains [8].

Simultaneously, cognitive decline exacerbates multimorbidity. Evidence suggests a bidirectional relationship between multimorbidity and cognition in older adults. This bidirectional association may be mediated by aging-related biological mechanisms, lifestyle factors, and adverse life events [9]. Therefore, coordinated efforts across biological, psychological, and social domains are necessary to jointly mitigate the bidirectional decline between multimorbidity and cognitive impairment and to enhance the health status and quality of life in older adults [10].

The establishment of a systemic low-grade inflammatory state is a hallmark of aging. Elevated levels of inflammatory factors have been shown to impair neuronal function and increase the risk of dementia [11]. Meanwhile, chronic diseases associated with aging further exacerbate peripheral and central inflammation, leading to worsened cognitive impairment [12]. Decreased cerebral glucose metabolism in the parietal–temporal, posterior cingulate, and frontal regions is considered a key feature of preclinical dementia and may represent early metabolic changes. Numerous diet-related metabolites, such as those involved in protein synthesis, energy metabolism, nitrogen waste management, and neuronal signaling, are essential for neurobiological functions [13]. This suggests that the metabolic health of the brain is influenced by dietary status. Increasing evidence indicates that dietary interventions, especially the Mediterranean diet, have substantial benefits for public health [14]. This diet has been shown to protect against chronic conditions, such as cardiovascular disease, cancer, type 2 diabetes, and dementia [15]. Its protective effects likely originate from its nutrient profile, which can directly and indirectly modulate brain aging by promoting neuronal plasticity and reducing inflammation and oxidative stress [16]. Another study specifically highlighted the importance of fat quality, carbohydrate quality, whole grain consumption, fish intake, and fruit and vegetable intake, which is regarded as the most supportive evidence for promoting brain health and reducing the risk of chronic disease [17]. However, previous studies on cognitive function in older adults with multimorbidity have focused on measuring only total scores and have not considered the relationship between specific symptoms and different foods. The relationship between diet, a key factor in multimorbidity that affects the health and well-being of older adults, and cognitive symptoms requires our close attention.

Network analysis methods have increasingly been applied in the field of psychosomatic disorders, enabling in-depth exploration of specific symptoms of various mental disorders and the visualization of relationships among complex variables [18]. Nodes and edges are the two core concepts in network analysis, in which the most influential symptom is regarded as the central node with numerous connections to other nodes, whereas bridge symptoms link separate symptom clusters [19]. These nodal features provide specific targets for intervention and preventive strategies. This study used a network approach to investigate the relationship between different food consumption and cognitive symptoms in older adults with multimorbidity by identifying key affective and bridging symptoms, thereby informing nutritional intervention strategies for cognitive functioning in this population.

## 2. Method

### 2.1. Source of Data and Participants

The cross-sectional data were derived from the 2017–2018 China Longitudinal Health Longevity Survey (CLHLS). Organized by the Center for Healthy Aging and Development Studies (CHADS) at Peking University, this survey represents the first nationwide longitudinal study of older adults in developing countries. From 1998 to 2018, the CLHLS was conducted seven times in half of the randomly selected counties/cities in 23 provinces in mainland China. All data from the CLHLS are publicly available at https://opendata.pku.edu.cn/dataverse/CHADS (accessed on 22 January 2025). Most participants were aged over 65 years, and detailed data were collected on the basic conditions, socioeconomic characteristics, health status, behavioral patterns, psychological characteristics, and dietary habits of older adults and their families [20]. The CLHLS received ethical approval from the Research Ethics Committee of Peking University (IRB00001052-13074), and written informed consent was obtained from all CLHLS participants prior to the survey.

**Figure 1:**
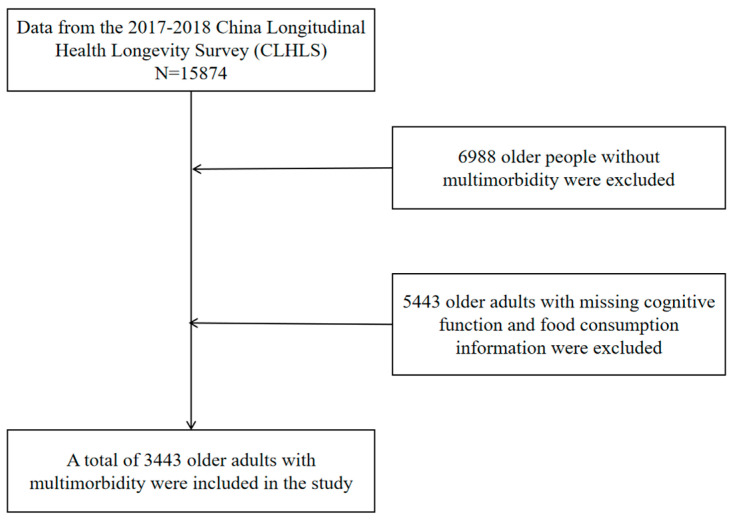
Flowchart of the participants.

Older adults who did not have multimorbidity and lacked cognitive function and food consumption information at the time of the survey were excluded. The final analysis included 3443 older adults. Figure 1 depicts the specific screening process.

### 2.2. Assessment of Food Consumption

In the CLHLS, information on food consumption was collected via a simplified Food Frequency Questionnaire (FFQ) included in the Lifestyle Module. This questionnaire gathered data on participants’ intake frequency of 13 specific food items, namely fresh fruits, fresh vegetables, meat, seafood, eggs, soybeans, salty vegetables, sugar, tea, garlic, nut products, mushrooms or algae, and dairy products. The consumption frequency of these foods was categorized into four levels: almost every day, quite often, occasionally, and rarely or never.

### 2.3. Cognitive Functions

This study employed the Chinese version of the Mini-Mental State Examination (MMSE) to assess the participants’ cognitive function. The Chinese MMSE has demonstrated good validity and reliability [21]. The MMSE in this version comprises 24 items across five domains: orientation (six items), memory (three items), attention and calculation (six items), recall (three items), and language (six items). In addition, item 6 was adapted as a food-naming task conducted for one minute, with one point awarded for each correctly named food item, up to a maximum of seven points. The total MMSE score ranges from 0 to 30, with one point awarded for each correct answer and zero points for incorrect responses. Cognitive impairment was defined using education-adjusted cutoffs: illiterate ≤ 17, primary education ≤ 20, and junior high school or higher ≤ 24.

### 2.4. Multimorbidity

Multimorbidity refers to the co-occurrence of two or more chronic diseases in an individual. Fifteen chronic conditions were used to measure multimorbidity, including “hypertension”, “diabetes”, “heart disease”, “stroke”, “bronchitis”, “tuberculosis”, “cataract”, “glaucoma”, “cancer”, “gastric ulcer”, “arthritis”, “cholecystitis”, “blood disease”, “chronic nephritis”, and “hepatitis”. We calculated the number of chronic conditions for each older adult based on these 15 conditions, and individuals with two or more chronic conditions were classified as having multimorbidity.

### 2.5. Covariates

Demographic variables collected included age (65–79 or ≥80 years), sex (male or female), marital status (other or married), education (illiterate, primary, or junior high school or higher), place of residence (rural or urban), health-related behaviors (including smoking no/yes, alcohol consumption no/yes, and exercise no/yes), and self-assessment of health status (poor, fair, or good).

### 2.6. Statistical Analysis

First, descriptive statistics for categorical variables were calculated using SPSS version 26.0, reporting frequencies and percentages. Second, network analysis was conducted using R (version 4.3.1). The R package “qgraph” was used to visualize the network model linking food consumption and cognitive function measures, with nodes representing variables and edges representing associations. Edges are positive (green) or negative (red), and the strength of the associations between nodes is indicated by edge thickness [22]. We applied the Gaussian Graphical Model (GGM) and used the graphical least absolute shrinkage and selection operator (glasso) process to construct regularized partial correlation networks. The Extended Bayesian Information Criterion (EBIC) was employed to select the optimal regularization parameters [23].

Node centrality can be measured using four metrics: strength, betweenness, closeness, and expected influence (EI). A higher centrality value indicates a more influential node in the network. This study employs expected influence (EI) to identify node centrality. This is because, in networks containing both positive and negative edges, EI outperforms the traditional strength centrality (a commonly used measure based on edge weights) [24]. The “networktools” package was used to compute the bridge centrality index and bridge strength to identify bridge symptoms in the network model.

In addition, the “bootnet” package was used to assess the accuracy of edge-weight estimates and the stability of centrality indices. A nonparametric bootstrap was employed to estimate the 95% confidence intervals (CIs) for edge weights, with narrower CIs indicating less volatility in the network. The stability of centrality indices was assessed using the correlation stability coefficient (CS-C): values greater than 0.25 indicate minimum stability; greater than 0.5 indicate sufficient stability; and greater than 0.70 indicate high stability [25].

## 3. Results

### 3.1. Descriptive Results

Table 1 presents the detailed demographics of the participants. The participants included 3443 older adults with multimorbidity, with a mean age of 79.44 (SD = 9.31) years: 1684 males and 1759 females.

Regarding social characteristics, 52.0% of the older adults with multimorbidity were married, 1205 (35.0%) were illiterate, and 2615 (76.0%) lived in rural areas. Concerning health-related behaviors, 43.4% of older adults with multimorbidity reported good health status, 83.6% smoked, 85.6% drank alcohol, and 56.5% exercised.

### 3.2. Analysis of Network Structure and Centrality Measures

**Figure 2:**
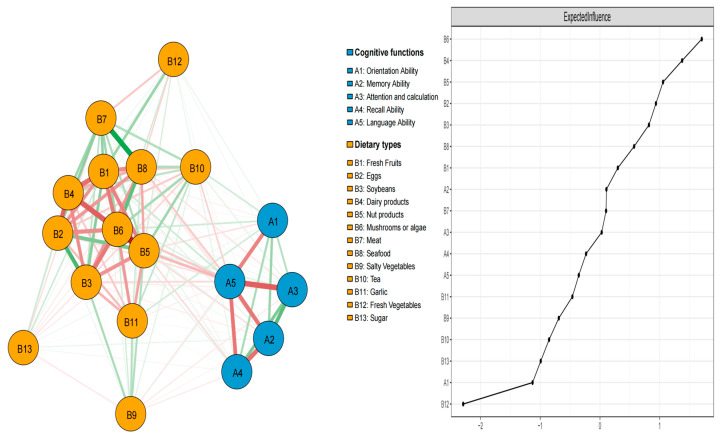
Network structure of food consumption and cognitive function in older adults with multimorbidity. Note: Green edges indicate positive associations, while red edges indicate negative associations.

The left subgraph in Figure 2 shows the network structure of food consumption and cognitive function, with a high network density of 0.52 (79/153) and an average edge weight of 0.03. Eighteen nodes yielded 153 potential edges, of which 79 edges had a non-zero weight (51.63%). The correlation matrix is presented in Table S1. The strength of the connections is depicted by the edge thickness and color intensity. Within the cognition subnetwork, the strongest association was between A3 (attention and calculation) and A5 (language ability), followed by A2 (memory ability) and A4 (recall ability). Within the food consumption subnetwork, the strongest association was between B5 (nut products) and B6 (mushrooms or algae), followed by B7 (meat) and B8 (seafood). Between food consumption and cognition function networks, A1 (orientation ability) and B1 (fresh fruits) were strongly associated, followed by A1 (orientation ability) and B12 (fresh vegetables); the the link between B1 (fresh fruits) and B12 (fresh vegetables) was the weakest.

The right subgraph of Figure 2 shows that within the cognition subnetwork, A2 (memory ability) had the highest expected influence (EI), whereas A1 (orientation ability) had the lowest EI. In the food consumption subnetwork, B6 (mushrooms or algae) had the highest EI, and B12 (fresh vegetables) had the lowest EI. In the full network model, B6 (mushrooms or algae), B4 (dairy products), and B5 (nut products) demonstrated higher EI, indicating that these nodes may be most strongly connected with other nodes, highlighting the central role of diet in the network. The centrality difference results (Figure S1) indicated that these nodes were statistically stronger than the others. Node B6 (mushrooms or algae) was the most EI in this network, whereas B12 (fresh vegetables) and A1 (orientation ability) had the lowest EI.

### 3.3. Bridge Network Structure and Bridge Strength Analysis

**Figure 3:**
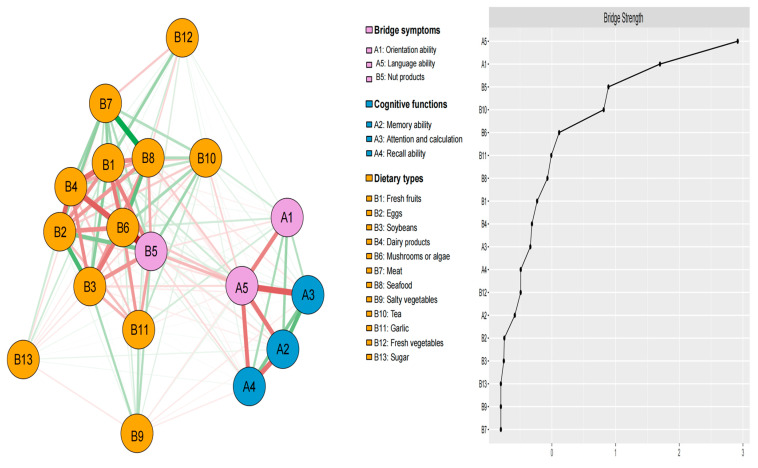
Bridge network structure of food consumption and cognitive function in older adults with multimorbidity. Note: Green edges indicate positive associations, while red edges indicate negative associations.

The right subplot of Figure 3 presents the bridge strength of the network. A5 (language ability) had the highest bridge strength, followed by A1 (orientation ability) and B5 (nut products), suggesting that these nodes may have acted as bridging nodes in the network. These high-bridge-centrality nodes were the most influential in the network and are referred to as bridge symptoms. The bridge node centrality difference results (Figure S2) revealed significant differences between bridge symptoms and edges, supporting plausible interpretations of the bridging network.

### 3.4. Network Stability

**Figure 4:**
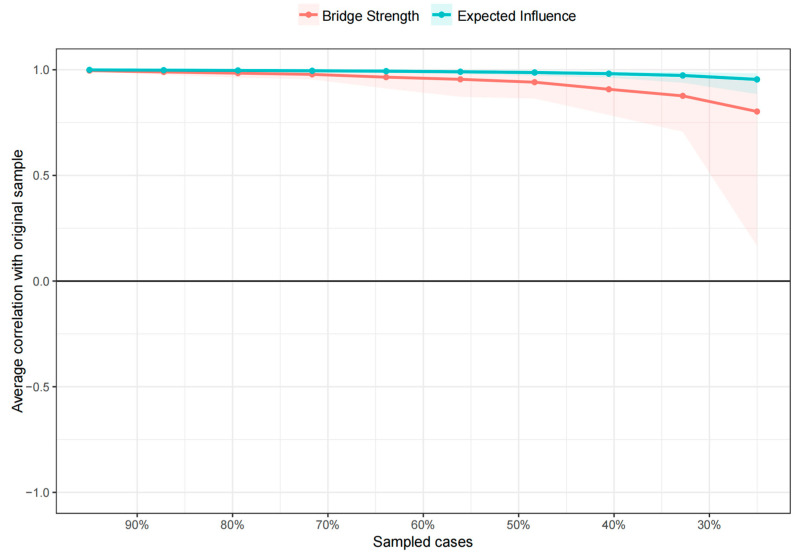
Stability of the expected influence and bridge strength of nodes. Note: The x-axis represents the percentage of the original sample size used in each step. The y-axis represents the average correlation between the centrality indices in the original network and the centrality indices that were re-estimated after excluding progressively increasing percentages of samples. The line indicates the correlation between the expected influence and bridging strength.

Figure S3 examines the accuracy of the marginals by bootstrap 95% confidence intervals, which are narrow, indicating good accuracy and reliable network model results. Figure 4 shows the results of the case-dropping bootstrap test. The CS coefficient of centrality EI was 0.75, indicating that the original network and the re-estimated order of centrality EI remained consistent at 0.75 when 75% or less of the sample size was decreased. The CS coefficient of the bridge strength was 0.75, indicating that the bridge strength was sufficiently stable.

## 4. Discussion

This study aimed to employ network analysis to investigate the relationship between food consumption and cognitive function in older adults with multimorbidity. Our findings indicate that each food consumption item and different cognitive nodes are generally closely interconnected. However, the correlations between food and cognition tend to be relatively modest. In the food consumption–cognitive function network model, B6 (mushrooms or algae), B4 (dairy products), and B5 (nut products) had the greatest influence. A5 (language ability), A1 (orientation ability), and B5 (nut products) were identified as key bridge symptoms linking food consumption and cognitive function within the network.

Each food consumption item was correlated with at least one cognitive function. However, significant heterogeneity was noted in the associations across different cognitive functions. For instance, B4 (dairy products) exhibited a positive correlation with A1 (orientation ability) and A3 (attention and calculation), indicating that dairy product consumption may enhance orientation, attention, and calculation abilities in elderly individuals with multimorbidity. In contrast, dairy consumption was negatively correlated with A2 (memory ability), A4 (recall ability), and A5 (language ability). This suggests that dairy consumption might also contribute to declines in recall and language abilities within this population. This may be due to differences in the types of dairy products and the demographic characteristics of the study samples [26]. Previous research has identified high meat consumption, such as red meat, as a risk factor for cognitive impairment among older adults [27]. However, our findings revealed that although B7 (meat) was negatively correlated with A1 (orientation ability), A2 (memory ability), A4 (recall ability), and A5 (language ability), it exhibited a positive correlation with A3 (attention and calculation). Thus, increasing meat consumption may enhance attention and calculation abilities in elderly individuals with multimorbidity. These findings imply that reliance on aggregate cognitive scores in prior research may have obscured or suppressed the detection of changes in specific cognitive domains, resulting in the loss of clinically valuable information [28].

B6 (mushrooms or algae) emerged as the most central node within the comprehensive network of older adults with multimorbidity. Central nodes significantly influence the overall network structure and function and may serve as critical determinants in the initiation, progression, and maintenance of cognitive processes [29]. Targeted interventions at central nodes may exert a substantial influence on improving overall cognitive function. Commonly edible mushroom species, such as mushrooms, fungus, snow fungus, and others, are rich in bioactive compounds, including beta-glucans, lovastatin, L-ergothioneine, ergosterol, and polyphenols. These compounds can help elderly individuals reduce cholesterol levels, mitigate oxidative stress and inflammation, and protect neurons from damage [30,31]. Edible algae, such as kelp and seaweed, contain various natural nutrients that effectively reduce inflammatory responses, alleviate oxidative stress, and regulate apoptosis [32]. Additionally, these algae are rich in vitamin B12, which stimulates neural pathways and helps prevent memory decline [33]. The characteristics of the bioactive compounds contained in these common edible mushrooms or algae make them naturally beneficial in preventing chronic diseases and cognitive impairment in humans.

Dairy and nuts are also central nodes in the network-wide model. Dairy products may impair or improve cognitive function in older adults with multimorbidity. This may be attributed to the fact that dairy products are rich in beneficial nutrients such as proteins, minerals, vitamins, and essential amino acids [34,35]. However, they also contain high levels of saturated fat, which may adversely affect cognitive function and chronic disease. Nuts are a key component of the Mediterranean diet, and their consumption may help prevent certain chronic diseases [36]. A randomized controlled trial indicates that long-term consumption of nuts may confer benefits to cerebrovascular function and enhance cognitive performance [37]. In summary, mushrooms, algae, dairy, and nut products may exert potential influence on chronic diseases and cognitive function. This makes them an ideal dietary choice for improving cognitive health in elderly people with multiple chronic conditions.

Cognitive status tends to be more susceptible in older adults with concomitant multimorbidity than in younger adults [38]. Bridge strength centrality guides the search for bridge symptoms in the food–cognition network of older adults with multimorbidity. Bridge symptoms are not only early markers of disease but may also have a ripple effect within and across the network. If the bridge symptom worsens, it affects other symptoms associated with it, which may lead to an overall decline in cognitive functioning [39]. Our study identified A5 (language ability), A1 (orientation ability), and B5 (nut products) as the bridge symptoms in this network model. Changes in language ability, indicated as the most important bridge symptom, often reflect disturbances in neural connectivity in multiple regions of the brain and are a key early manifestation of cognitive impairment in older adults with multimorbidity [40]. Language ability forms the foundation of cognitive processes, and language ability plays a key role in the encoding, retrieval, storage, and expression of information; language disorders not only affect communication and social functioning but also further exacerbate cognitive deterioration [41]. Evidence suggests that vestibular function in the brain is closely related to cognitive orientation, and that the vestibular–visual–proprioceptive system synergizes to form the neural basis of spatial perception and localization [42]. Vestibular signals project through the vestibular nuclei, thalamus, and cortex to areas such as the parahippocampal gyrus, medial prefrontal cortex, and parietal cortex to support spatial memory, path navigation, and spatial localization of the body [43]. Vestibular function declines with age, and accurate orientation depends on intact vestibular signaling and intact cognitive function [44]. Multimorbidity may exacerbate language and orientation impairments in older adults. Nut products serve as another potential bridge symptom. Nut products, rich in unsaturated fatty acids, may help prevent cognitive decline by modulating insulin sensitivity and reducing inflammation [45,46]. Additionally, the nutrients contained in nut products have a significant protective effect against multimorbidity [47]. Therefore, interventions for language and orientation in older adults with multimorbidity can help maintain the relevant neural pathways, delay the development of cognitive deficits, and prevent the development of dementia. At the same time, combined with a targeted nutritional intervention program, it can help with neuronal repair and remodeling.

Our study has two main strengths. On the one hand, we used a standardized assessment tool, network analysis, to identify intervention targets in food consumption–cognition function networks, providing a scientific basis for individualized interventions for cognition in older adults with multimorbidity. However, the data were obtained from a large cohort survey in China with a representative and stable sample. Although this study provides new profiles of the relationship between food consumption and specific cognitive symptoms, it has some limitations. First, our cross-sectional design could not demonstrate a causal relationship between food consumption and cognitive function. Second, there may be some recall bias in the use of self-reported recall to collect data on food consumption and cognitive function. Finally, although the study sample was nationally representative, it still has limited applicability to certain regions or specific populations. Future validation in different populations is needed to enhance the general applicability and generalization value of the study.

## 5. Conclusions

In summary, this study highlights the key characteristics of the food consumption and cognitive function network in older adults with multimorbidity. This study identified B6 (mushrooms or algae), B4 (dairy products), and B5 (nut products) as central nodes in the food consumption and cognitive function network of older adults with multimorbidity. Additionally, A5 (language ability), A1 (orientation ability), and B5 (nut products) were identified as bridge symptoms connecting food consumption and cognitive function networks. These findings highlight the potential for targeted food consumption interventions in older adults with multimorbidity, making them a key focus for improving cognitive function in this population.

## Figures and Tables

**Table 1:** Basic characteristics of participants.

Variables	Number (%)
Age	
65–79	1853 (53.8)
≥80	1590 (46.2)
Sex	
Male	1684 (48.9)
Female	1759 (51.1)
Marital status	
Married	1790 (52.0)
Other	1653 (48.0)
Education	
Illiterate	1205 (35.0)
Primary	904 (26.3)
Junior high school or higher	1334 (38.7)
Place of residence	
Rural	2615 (76.0)
Urban	828 (24.0)
Self-assessment of health status	
Good	1495 (43.4)
Fair	1412 (41.0)
Poor	536 (15.6)
Smoking	
Yes	2878 (83.6)
No	565 (16.4)
Alcohol consumption	
Yes	2947 (85.6)
No	496 (14.4)
Exercise	
Yes	1945 (56.5)
No	1498 (43.5)

## Data Availability

The raw data supporting the conclusions of this article are available at https://opendata.pku.edu.cn/dataverse/CHADS (accessed on 22 January 2025).

## Supplementary Materials

The following supporting information can be downloaded at https://www.mdpi.com/article/10.3390/nu17172767/s1, Figure S1: Bootstrapped difference test for EI of food consumption and cognitive function.; Figure S2: Bootstrapped difference test for bridge strength of food consumption and cognitive function; Figure S3: Bootstrapped confidence intervals of edge weights; Table S1: Weighted adjacency matrix of the food consumption and cognitive function.
