# Supplementary material for: A Network Analysis of Food Intake and Cognitive Function in Older Adults with Multimorbidity: A National Cross-Sectional Study
Source: Nutrients. 2025 Aug 27;17(17):2767. doi: 10.3390/nu17172767 (PMC12430258; doi:10.3390/nu17172767)
Supplement: Supplementary file 1 [file nutrients-17-02767-s001.zip › nutrients-3786507-supplementary.pdf]

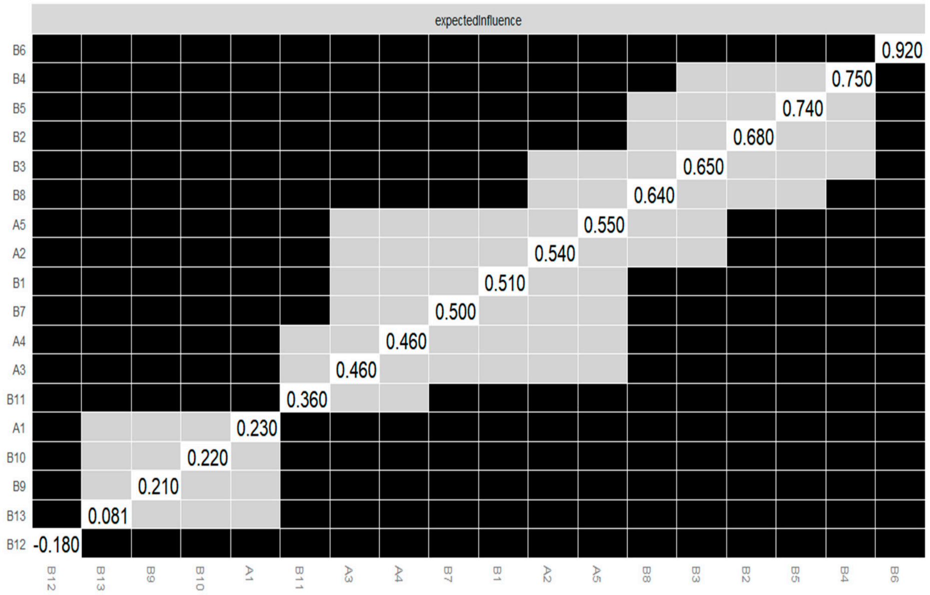

Figure S1. Bootstrapped difference test for EI of food consumption and cognitive function. Note: Gray boxes indicate that there are no significant differences between nodes' expected influences, while black boxes indicate that there are significant differences between nodes' expected influences. The numbers in the white boxes (i.e., diagonal lines) indicate the numerical values of the nodes' expected influences.

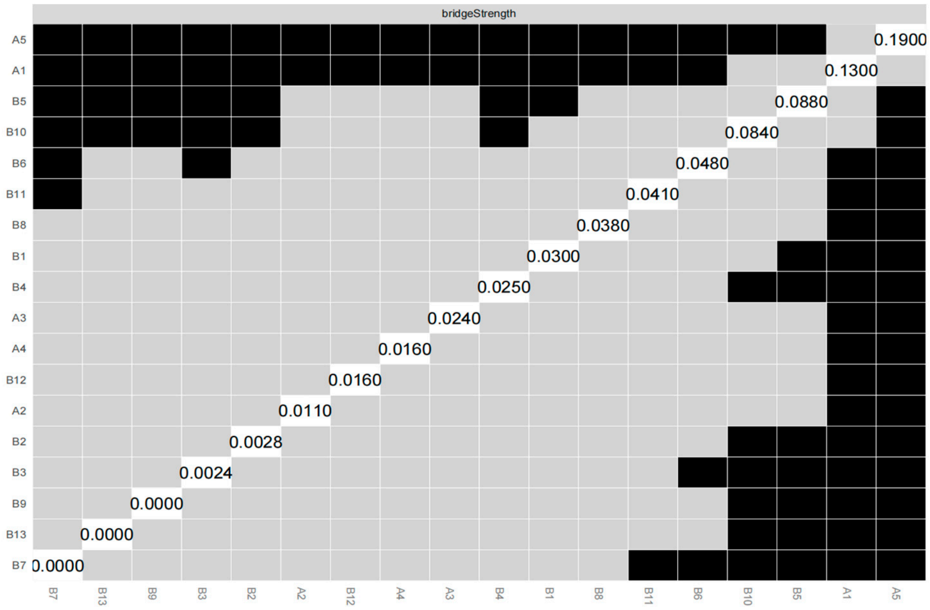

Figure S2. Bootstrapped difference test for bridge strength of food consumption and cognitive function. Note: Gray boxes indicate that there are no significant differences between nodes' bridge strength, while black boxes indicate that there are significant differences between nodes' bridge strength. The numbers in the white boxes (i.e., diagonal lines) indicate the numerical values of the nodes'bridge strength.

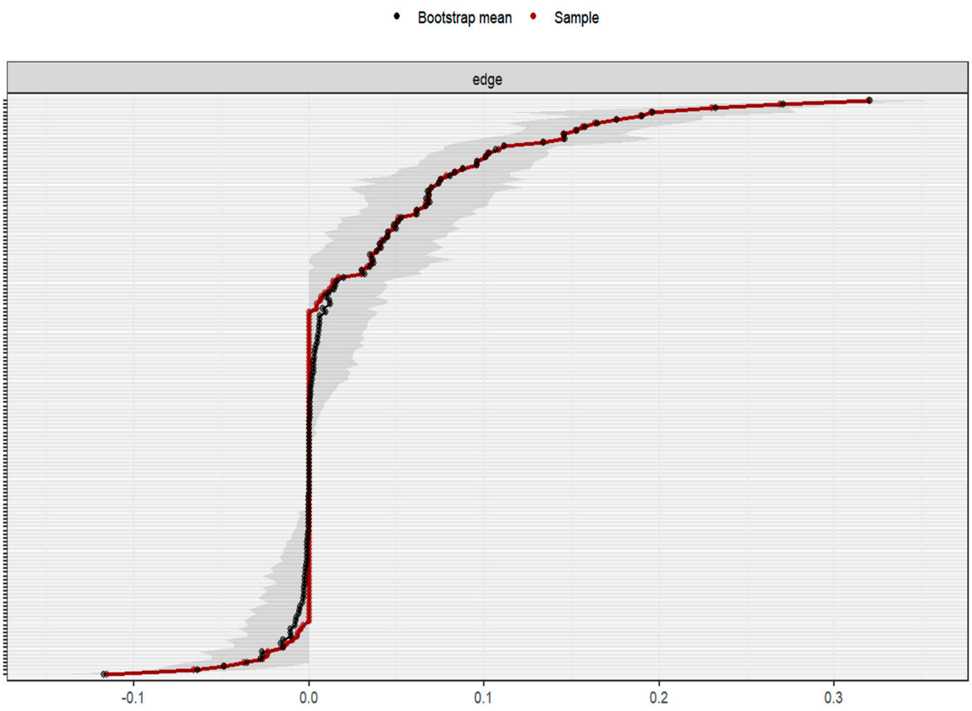

Figure S3. Bootstrapped confidence intervals of edge weights. Note: The red line indicates the edge estimated in the sample, and the black dot indicates the bootstrap mean for that edge. Gray areas represent 95% bootstrap confidence intervals for the edge, with narrower gray lines indicating more accurate estimates of the edge.

Table S1. Weighted adjacency matrix of the food consumption and cognitive function.

	A1	A2	A3	A4	A5	B1	B2	B3	B4	B5	B6	B7	B8	B9	B10	B11	B12	B13
A1	1																	
A2	0.177	1																
A3	0.132	0.271	1															
A4	0.151	0.307	0.219	1														
A5	0.267	0.291	0.341	0.296	1													
B1	-0.030	-0.047	-0.022	-0.041	-0.090	1												
B2	0.003	-0.023	-0.004	-0.002	-0.068	0.231	1											
B3	-0.009	-0.039	-0.043	-0.042	-0.082	0.207	0.291	1										
B4	0.020	-0.012	0.030	-0.013	-0.061	0.308	0.333	0.248	1									
B5	-0.066	-0.068	-0.054	-0.080	-0.153	0.271	0.226	0.232	0.302	1								
B6	-0.084	-0.048	-0.013	-0.047	-0.120	0.283	0.247	0.287	0.340	0.470	1							
B7	-0.017	-0.047	0.014	-0.034	-0.038	0.181	0.197	0.118	0.118	0.173	0.142	1						
B8	-0.008	-0.054	-0.057	0.009	-0.113	0.245	0.222	0.223	0.175	0.188	0.263	0.362	1					
B9	0.051	-0.026	-0.048	-0.044	-0.045	0.041	0.038	0.150	0.015	0.141	0.082	-0.001	0.053	1				
B10	-0.084	-0.024	-0.047	-0.047	-0.099	0.113	0.110	0.088	0.117	0.170	0.156	0.136	0.140	0.056	1			
B11	-0.080	0.016	0.001	-0.015	-0.039	0.081	0.144	0.162	0.120	0.198	0.221	0.125	0.089	0.134	0.111	1		
B12	0.040	0.011	-0.028	0.030	0.026	-0.179	-0.044	0.006	-0.037	-0.062	-0.084	-0.117	-0.093	0.003	-0.041	-0.069	1	
B13	0.029	0.038	0.013	0.023	0.007	0.090	0.061	0.057	0.090	0.043	0.041	0.073	0.047	0.053	0.039	-0.001	0.031	1
